# Noninvasive ventilation in acute respiratory failure due to H1N1 influenza: A word of caution

**DOI:** 10.4103/0970-2113.80340

**Published:** 2011

**Authors:** Akashdeep Singh, Jaspreet Singh

**Affiliations:** *Department of Pulmonary and Critical Care Medicine, Christian Medical College and Hospital, Ludhiana, Punjab, India. E-mail: drsinghakashdeep@gmail.com*; 1 *Department of Anesthesiology, Peninsula Regional Medical Center, Salisbury, MD 21801, USA*

Sir,

The case of acute respiratory failure due to H1N1 influenza managed by noninvasive ventilation (NIV) described by Mohapatra *et al*.[1] is very interesting, but there are few technical issues which need to be discussed.

First, the use of NIV in hypoxemic respiratory failure is controversial, and the etiology of hypoxemia appears to be an important determinant of its success. A meta-analysis[2] suggests that noninvasive positive-pressure ventilation (NPPV) does not decrease the need for intubation, so there is not enough evidence to support its use in acute respiratory distress syndrome. There are only a few patients with H1N1-related respiratory failure who seem to benefit from NIV alone, so it should be reserved for patients with milder disease. Guidelines endorsed by the European Respiratory Society and European Society of Intensive Care Medicine[3] conclude that, as a general rule, NIV should not be recommended as an alternative to invasive ventilation in patients affected by H1N1.

Second, NIV is a potential aerosol-generating device. In this regard, the deliberate leakage via the exhalation ports may generate droplet nuclei and disperse infective aerosols through the evaporation of the water content of respiratory droplets resulting in a superspreading event.[4]

A case–control study[5] involving 124 medical wards in 26 hospitals in Guangzhou and Hong Kong has identified SARS patients requiring NPPV as independent risk factors for spreading nosocomial outbreaks of SARS.

Keeping these two facts in mind, it is very difficult to justify NIV usage in patients with acute respiratory failure due to H1N1 influenza.
